# 

**Published:** 1916-10

**Authors:** 


					﻿PUBLISHED MONTHLY.	rpz\ Xrp2\	SUBSCRIPTION $1.00
journal-reookhF
q d "
! Atlanta Medical and Surgical Journal, Established 1855.	)	/ q i v
Mfd Ypar
and Southern Medical Record, Established 1870.	\ "<->• U I VOI
Vol. LXIII	Atlanta, Ga., October, 1916	No. 7
				

